# Growth Biocontrol of Foodborne Pathogens and Spoilage Microorganisms of Food by Polish Propolis Extracts

**DOI:** 10.3390/molecules24162965

**Published:** 2019-08-15

**Authors:** Katarzyna Pobiega, Karolina Kraśniewska, Jarosław L. Przybył, Katarzyna Bączek, Joanna Żubernik, Dorota Witrowa-Rajchert, Małgorzata Gniewosz

**Affiliations:** 1 Division of Food Biotechnology and Microbiology, Department of Biotechnology, Microbiology and Food Evaluation, Faculty of Food Sciences, Warsaw University of Life Sciences SGGW, Nowoursynowska 159c, 02-776 Warsaw, Poland; 2 Laboratory of New Herbal Products, Department of Vegetable and Medicinal Plants, Faculty of Horticulture, Biotechnology and Landscape Architecture, Warsaw University of Life Sciences SGGW, Nowoursynowska 159c, 02-776 Warsaw, Poland; 3 Department of Food Engineering and Process Management, Faculty of Food Sciences, Warsaw University of Life Sciences SGGW, Nowoursynowska 159c, 02-776 Warsaw, Poland

**Keywords:** propolis, antimicrobial activity, antioxidant activity, time-kill, bacteria, yeast, mold

## Abstract

Propolis is a natural mixture produced by bees from plant resin substances. This study focuses on the general characteristics of five samples of Polish extract propolis originating from agricultural areas. Chemical composition with high performance liquid chromatography‒diode array detector method, total content of flavonoids and polyphenols, and antioxidative activity were determined in the ethanol extracts of propolis (EEP) samples. Minimum inhibitory concentration (MIC), minimum bactericidal/fungicidal concentration (MBC/MFC) and time-kill curves were studied for foodborne pathogens and food spoilage microorganisms. In EEPs the predominant flavonoid compounds were pinocembrin, chrysin, pinobanksin, apigenin, and kaempferol and the predominant phenolic acids were p-coumaric acid, ferulic acid, and caffeic acid. A strong antioxidative action of propolis in vitro was observed (IC_50_ for DPPH radical was at the level of 0.9–2.1 µg/mL). EEPs had MIC values for bacteria in the range of 1–16 mg/mL, whereas MIC for fungi ranged from 2 to 32 mg/mL. Extract of propolis originating from southern Poland was distinguished by higher content of bioactive components, and stronger antioxidative and antimicrobial activity than EPPs from the remaining areas of Poland. The results indicate the possibility of applying ethanol extracts from Polish propolis to protect food against microbiological spoilage.

## 1. Introduction

Foodborne pathogens constitute a serious hazard for public health around the world. Statistical data show that known foodborne pathogens cause annually 9.4 million diseases in the United States and over 350,000 in the European Union member states [1,2]. Food contaminated with foodborne pathogens or their toxins constitutes the cause for these diseases [1]. One of the efficient methods of the elimination of pathogen development in foods is chemical preservation consisting of adding conventional preservatives to foods, i.e., benzoic acid, sodium benzoate, propionic acid, sorbate, hydrogen peroxide, sulfides (hydrogen sulfite, metabisulfite), nitrite, and nitrate [3]. Despite the considerable efficiency of their impact on microorganisms, consumers increasingly commonly abandon chemically preserved foods due to their potentially negative impact on health [4]. Conventional preservatives may cause asthma, abdominal pain, nausea, diarrhea, hives, itching, allergies, lung irritation, and even cancers [5,6,7]; giving rise to the need for the search for natural antimicrobial substances, which would efficiently replace chemical preservatives. To this end, essential oils and plant extracts have been studied for several decades, indicating their usability in the prolongation of raw material and food products shelf-life [8,9,10,11]. 

Propolis is a natural mixture of resin substances collected by bees [12]. From ancient times, humans have been using propolis in various fields, primarily in traditional medicine; thus, it is well-known worldwide as a natural product improving health status and preventing diseases [13,14,15]. The pharmacological effect of propolis stems from its chemical composition. Thanks to the presence of numerous phenolic compounds and their derivatives in the composition, propolis demonstrates antibacterial activity [16,17]. Certain derivatives of cinnamic acid and flavonoids are responsible for energy splitting in the cytoplasmic membrane, resulting in inhibition of bacterial movement, which contributes to the antibacterial effect [18]. Depending on the chemical structure, the flavonoids present in propolis are classified among flavones, flavanones, isoflavones, chalcones, dihydrochalcones, isoflavanes, isodihydroxyflavones, and neoflavonoids, and the relatively rare flavonoid C-glycosides and isorhamnetin 3-o-rutinoside [19]. Phenols comprise a large group of compounds present in propolis. Phenols are organic compounds, derivatives of aromatic hydrocarbons, containing hydroxy groups bonded directly with the aromatic ring. In its composition, propolis primarily contains phenolic acids, aromatic esters, alcohol phenols, aldehyde phenols, coumarins, and ketophenols. The most common phenolic acids include caffeic acid, cinnamic acid, ferulic acid, and *p*-coumaric acid [20].

The chemical composition of propolis is variable and depends on numerous environmental factors, such as climate, vegetation of the given region, propolis harvest season, and its geographic origin [21]. According to Bankova [22] the following propolis types can be distinguished by plant origin and propolis chemical composition: Poplar propolis, Birch propolis, green (Alecrim) propolis, red (*Clusia*) propolis, Pacific propolis, and Canarian propolis. Propolis types are characterized by differing chemical composition, which determines their biological properties [14,22]. Algerian propolis demonstrates remarkable activity against foodborne pathogens, including Gram-positive bacteria *Bacillus cereus* and *Staphylococcus aureus*, and Korean propolis demonstrates strong inhibitory activity, in particular to vegetative cells of *B. cereus*. Ethanol extract of Turkish propolis exhibited strong antilisterial activity and slightly less pronounced activity against *Salmonella* Enteritidis [23,24,25]. 

In order to reduce the count or completely eliminate foodborne pathogens and saprophytic microbiota in foods, propolis extracts can be added directly to food or applied superficially. Immersing foods in propolis extracts or the application of specifically prepared coatings containing propolis extracts is a method restricting the characteristic taste and aroma of propolis, which may have a negative influence on the sensory properties of food to which it is added [21].

Previous studies on the antimicrobial activity of Polish propolis extracts were mainly related to pathogenic bacteria, e.g., *S. aureus, E. coli*, yeast *Candida spp*. and some types of mold [26,27,28,29,30]. Studies on the growth biocontrol of foodborne pathogens by Polish propolis are scarce. Thus, the objective of the present study was to compare the antimicrobial and antioxidative activity of five samples of Polish propolis, originating from different agricultural regions of Poland. Based on the propolis samples, ethanol extracts were prepared, which were subsequently tested for their chemical composition and antioxidative and antimicrobial activity.

## 2. Results

### 2.1. HPLC-DAD Validation Parameters

Propolis is a very complex matrix consisting of many groups of phytochemicals including many phenolic compounds. A high efficiency column, characterized by good chromatographic resolution enabling single, narrow peaks, was required for separation, accurate identification, and quantitation of each compound creating such a multicomponent mixture. Separation was achieved using a C18 reversed-phase column filled up with 2.6-μm particles with a solid core and porous outer layer, which provided high throughput. This kind of stationary phase enables high resolution, with the application of short column length (100 × 4.6 mm), a flow rate of 2.0 mL/min, oven temperature of 45 °C as well as proper gradient elution resulting in short retention times and narrow peaks. Moreover, narrow peaks translate to an increase in signal intensity, allowing for better determination of low-concentration constituents (sensitivity increase). Unfortunately, the large number of analytes extended the analysis time to 40 min and increased the mobile phase usage. As a result of the work carried out, a method allowing the separation and determination of 14 flavonoids, 12 phenolic acids and cinnamyl alcohol was constructed and validated. The method was characterized by acceptable precision (<2.5%), very good linearity (r > 0.999) over a wide range, good sensitivity, and satisfactory accuracy (Table 1). 

### 2.2. Composition of Phenolic Compounds and Chemical Profile of EEPs

**Figure 1:**
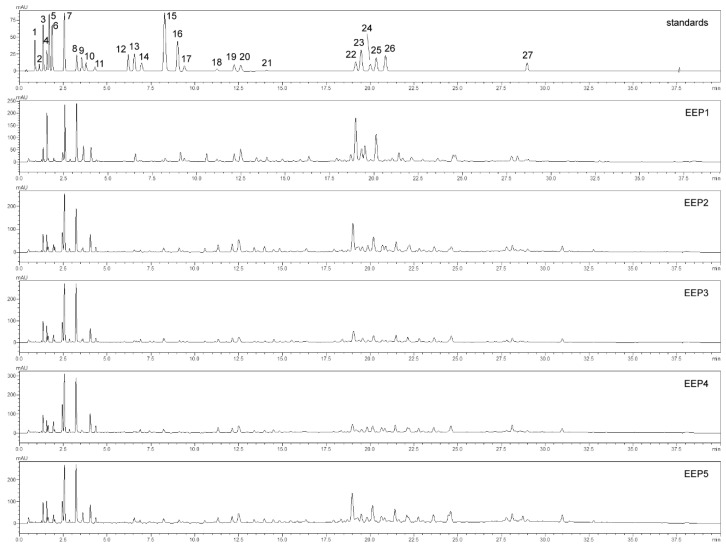
HPLC-DAD profile of ethanol extracts of propolis (1: protocatechuic acid, 2: (+)-catechin, 3: 4-hydroxybenzoic acid, 4: caffeic acid, 5: vanillic acid, 6: syringic acid, 7: p-coumaric acid, 8: ferulic acid, 9: ellagic acid dehydrate, 10: quercetin-3-O-rutinoside, 11: cichoric acid, 12: apiin, 13: dimethyl caffeic acid, 14: cinnamyl alcohol, 15: cinnamic acid, 16: 4-methoxycinnamic acid, 17: quercetin, 18: pinobanksin, 19: apigenin, 20: kaempferol, 21: isorhamnetin, 22: chrysin, 23: pinocembrin, 24: acacetin, 25: galangin, 26: oroxylin; 27: (+/−)-pinostrobin).

HPLC analysis of the tested propolis extracts enabled identification of a total of 27 phenolic compounds classified among flavonoids and phenolic acids (Figure 1, Table 2). The Total Flavonoid Content (TFC) in EEPs was on average between 1753.09 and 10196.09 mg/mL, and phenolic acids in the range between 4371.53 and 6326.85 mg/mL. The flavonoid group included chemical compounds classified among flavanones, flavanonoles, flavones, flavonols, and flavans. The predominant flavonoid compounds were pinocembrin and 5,7-dihydroxyflavone (chrysin), pinobanksin, apigenin, and kaempferol. The predominant phenolic acids were 4-hydroxycinnamic acid (*p*-coumaric acid), 4-hydroxy-3-methoxycinnamic acid (ferulic acid), and 3,4-dihydroxycinnamic acid (caffeic acid).

Propolis extracts differed in the content of chemical components. EEP1 was the most flavonoid-rich propolis extract, which contained a particularly high number of flavones with regard to the remaining extracts. EEP1 contained the highest amounts of chrysin, pinocembrin, galangin, pinobanksin, and phenolic acids: caffeic acid and dimethyl caffeic acid. In turn, EEP2 contained considerably higher amounts of flavanones than the remaining propolis extracts and it was characterized by a higher average content of pinocembrin and pinostrobin. EEP3 and EEP4 extracts were characterized by higher average contents of phenolic acids: *p*-coumaric acid, ferulic acid, and vanillic acid. EEP5 had lower average contents of all phytochemicals than the remaining tested propolis extracts.

### 2.3. EEPs Antioxidative Properties

The total polyphenols content (TPC) in samples of propolis extracts ranged from 52.65 to 100.29 mg/g, and the total flavonoid content (TFC) ranged from 8.23 to 15.55 mg/g (Table 3). Antioxidative activity of propolis extracts assessed with the use of DPPH and ABTS tests demonstrated that IC_50_ ranged, respectively, from 0.93 to 2.08 μg /mL and from 0.31 to 0.60 μg/mL. Considering the obtained results, all propolis extracts had strong antioxidative activity. The highest TPC content characterized EEP1, which was distinguished by its higher antioxidant activity in DPPH tests. A negative correlation between TPC and DPPH scavenging activity was observed (r^2^ = −0.98, *p* < 0.05) and an average negative correlation between TPC and ABTS radical activity was noted (r^2^ = −0.43, *p* < 0.05). No correlation was found between TFC and the antioxidative properties of the tested extracts (r^2^ DPPH = −0.38, r^2^ ABTS = 0.23, *p* < 0.05). 

### 2.4. Antibacterial Activity of EEPs

Assessment of the antibacterial activity of EEP was performed using standardized techniques, determining MIC and MBC for 11 foodborne pathogens. Results of MIC and MBC are presented in Table 4. All the tested bacterial strains were sensitive toward EEPs. The strongest effect against Gram-positive bacteria was demonstrated by EEP1, whose MIC values ranged between 1 and 4 mg/mL. The remaining extracts had moderate efficacy with MIC values ranging from 2 to 8 mg/mL. *B. cereus* (MIC 2–4 mg/mL) and *S. aureus* (MIC 1–8 mg/mL) were the most sensitive to the effect of propolis extracts, whereas lower sensitivity was exhibited by *L. monocytogenes* and *E. faecalis* (MIC 4–8 mg/mL). Propolis extracts demonstrated bactericidal effect in high concentrations (MBC ≥ 8 mg/mL), with the exception of EEP1 against *S. aureus* (MBC 4 mg/mL).

Extracts of Polish propolis exhibited moderate or poor effect toward Gram-negative bacteria. EEP1 demonstrated a bacteriostatic effect in the range between 2 and 16 mg/mL, and the remaining propolis extracts from 8 to 16 mg/mL. *E. coli* O157 was the most resistant strain (MIC 16 mg/mL). The MBC values range for all propolis extracts was from 8 to 32 mg/mL. 

Table 5 compares the antibacterial activity of propolis extracts in the tested concentration range. All tested extracts did not exhibit bacteriostatic effect at a concentration of 0.5 mg/mL. EEP1 demonstrated bacteriostatic effect at 1 mg/mL concentration, but only toward 9% of strains. At higher concentrations (2 and 4 mg/mL), EEP1 showed a wider spectrum of bacteriostatic effects than the remaining propolis extracts. All propolis extracts inhibited the growth of all the tested strains only at concentrations of 16 and 32 mg/mL.

**Figure 2:**
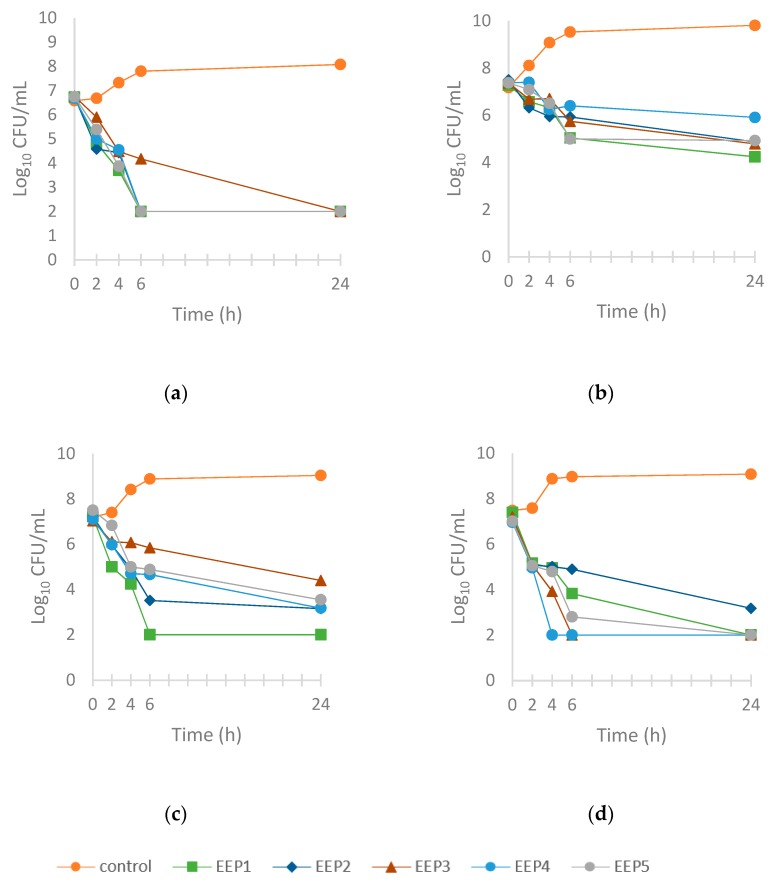
Time-kill curves shows of ethanol extracts of propolis against bacteria. (**a**) *S. aureus*, (**b**) *L. monocytogenes*, (**c**) *S.* Enteritidis, (**d**) *E. coli* O157.

Figure 2 represents the growth control (without propolis extract) and the time-kill curves (with propolis extract) for two Gram-positive bacterial strains (*S. aureus* and *L. monocytogenes*) and two Gram-negative bacterial strains (*S.* Enteritidis and *E. coli*). This test was used to analyze the dynamics of bacterial growth after the application of propolis extracts used in MICs. 

Considering the obtained results, inhibition of growth of test bacteria strains was observed after 2 h of propolis extract action. After 4 h the growth of *S. aureus* was inhibited to a greater degree by EEP5 and EEP1, which reduced viable counts by 3.4–3.6 log CFU/mL (Figure 2a). EEP2, EEP3, and EEP4 exhibited less pronounced effects with a decrease of viable counts by 2.8–2.9 log CFU/mL as compared with the initial count. After 6 h propolis extracts demonstrated bactericidal effect with maximum reduction of viable counts of *S. aureus* by 5.8 log CFU/mL, with the exception of EEP3. 

The inhibitory effect of propolis extracts on *L. monocytogenes* was more variable (Figure 2b). After 4 h only EEP2 reduced the variable count by 3.1 log CFU/mL. However, after 24 h EEP1 reduced the viable count by 5.6 log CFU/mL, and the following extracts by 4.9–5.0 log CFU/mL (EEP2, EEP3, and EEP5) and by 3.9 log CFU/mL (EEP4).

The strongest growth inhibiting effect on *S.* Enteritidis was demonstrated by EEP1 (Figure 2c). After 4 h it reduced the variable count of *S.* Enteritidis by 4.2 log CFU/mL, and after 24 h by 6.9 log CFU/mL relative to the initial count. The least pronounced growth inhibiting effect on *S.* Enteritidis was demonstrated by EEP3. 

Growth of *E. coli* was inhibited most significantly by EEP4 (Figure 2d). After 4 h the variable count was reduced by 6.9 log CFU/mL. In 2–6 h the effect of the remaining propolis extracts was less pronounced, but after 24 h they reduced the viable counts by approximately 5 log CFU/mL. 

### 2.5. Antifungal Activity of EEPs

Table 6 presents the MIC and MFC values of the tested propolis extracts. Low MIC values against the tested fungi were reported for EEP2 (2–8 mg/mL) and EEP1 (2–16 mg/mL). Fungicidal effect of EEP2 was observed in the MFC range of 2–32 mg/mL. Similar values of MFC in the range of 4–32 mg/mL was demonstrated by EEP1 depending on the test strain. EEP3 and EEP4 demonstrated fungistatic and fungicidal effect in the same concentration ranges, i.e., MIC/MFC from 4 to 32 mg/mL. Similar MIC/MFC values were found for EEP5 (2–32 mg/mL). Among yeasts, *R. mucilaginosa* was most sensitive to the effect of all propolis extracts (MIC 4–8 mg/mL). The growth of *C. albicans* was subject to stronger inhibition by EEP1 and EEP2 (MIC 2 and 4 mg/mL, respectively). In turn, among molds, *C. gloeosporioides* and *F. solani* were most sensitive to the effect of propolis extracts (MIC 2–4 mg/mL).

Table 7 compares the fungicidal activity of propolis extracts in the tested concentration range. None of the tested extracts exhibited any inhibitory effect to fungal growth at concentrations of 0.5 and 1 mg/mL. At a concentration of 2 mg/mL, three extracts (EEP1, EEP2, and EEP5) demonstrated fungicidal activity. In the concentration ranging between 2 and 16 mg/mL, EEP2 had a wider spectrum of action, followed by EEP1. All the tested fungi strains were inhibited by propolis extracts only at the concentration of 32 mg/mL.

**Figure 3:**
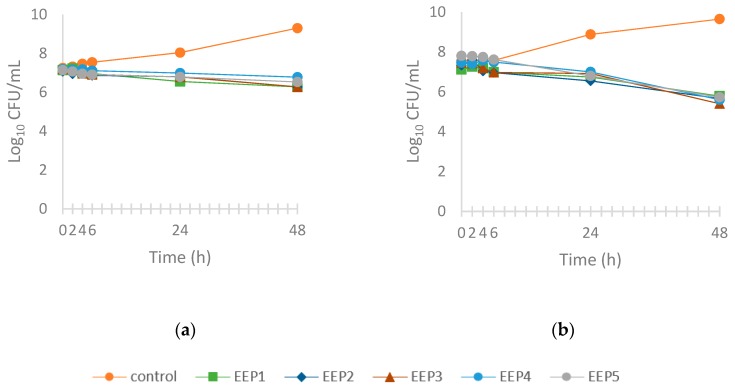
Time-kill curves of ethanol extracts of propolis against yeast. (**a**) *R. mucilaginosa*, (**b**) *C. albicans*.

Figure 3 demonstrates growth control (without propolis extract) and the time-kill curves (with propolis extract) of two yeast strains: *R. mucilaginosa* and *C. albicans*. All EEPs demonstrated similar action against *C. albicans* and *R. mucilaginosa*. Within the first 6 h no inhibitory effect on this yeast growth was observed. After 24 h of EEP1 action, reduction of viable count of *R. mucilaginosa* by 1.5 log CFU/mL was observed, whereas EEP2, EEP3, EEP4, and EEP5 inhibited yeast growth by 1.1–1.2 log CFU/mL (Figure 3a). After 48 h, reduction of viable count of *R. mucilaginosa* by EEP1, EEP2, and EEP3 by 3.0 log CFU/mL, and by EEP5 and EEP4 by 2.8 and 2.5 log CFU/mL, respectively, was observed. Equal effect of all propolis extracts was determined for *C. albicans* (Figure 3b). After 24 h propolis extracts inhibited *C. albicans* by 1.9–2.2 log CFU/mL, whereas after 48 h the variable cell was reduced by 3.9–4.2 log CFU/mL. This section may be divided by subheadings. It should provide a concise and precise description of the experimental results, their interpretation as well as the experimental conclusions that can be drawn.

## 3. Discussion

**Figure 4:**
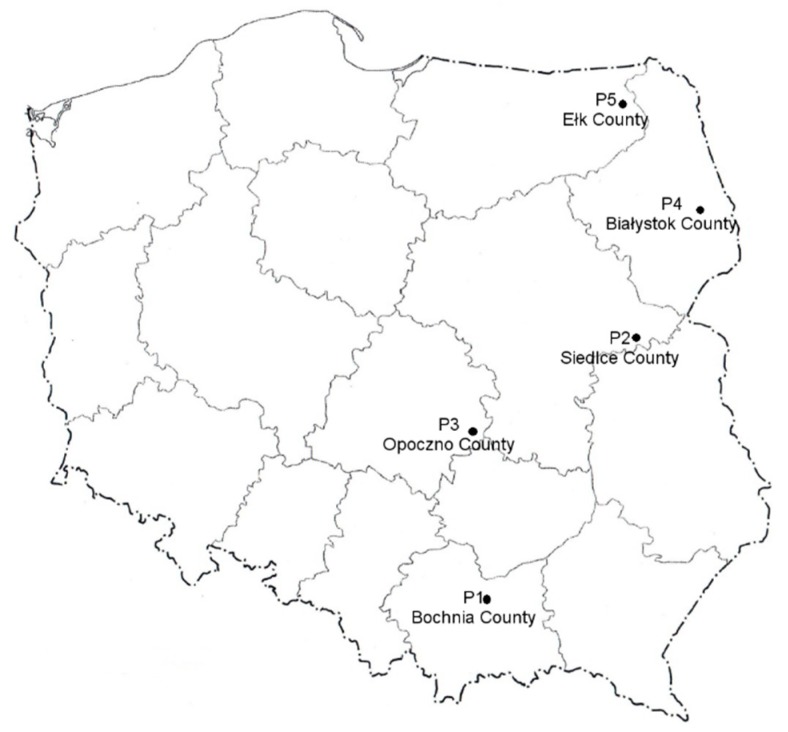
Polish map showing the location of sampling propolis from five different Polish counties.

In this study, we examined the chemical composition and biological properties of propolis originating from typical agricultural areas of Poland (Figure 4). Propolis from various areas of the world differs in appearance, chemical composition, and biological properties [22,31]. Due to its geographical location, Polish propolis is classified among European propolis. Such types of propolis are obtained by bees from vegetation typical of the Polish region, mixed forests with poplars, birches, alders, and conifers. 

Based on the detailed characteristics of the chemical composition of samples of Polish propolis (Table 2), it is evident that all samples contained phenolic compounds, including flavonoids and phenolic acids. Earlier studies reported that from the buds of *Populus nigra* and propolis collected from southern Poland, bioactive phytochemicals were isolated, which are the most common precursors of the “poplar type” of propolis typical of the temperate zone, i.e., pinostrobin, pinocembrin, pinobanksin-3-*O*-acetate, chrysin, and methyl-butenyl-*p*-coumarate and *p*-coumaric acid [22,27,32,33,34,35]. Flavonoids were also predominant in propolis from Serbia, Bulgaria, Bosnia, and Herzegovina, originating from *P. nigra* and *P.* x *euramericana* buds. Higher amounts of pinocembrin, chrysin, and pinostrobin were found in Serbian propolis [36,37]. Samples of Polish propolis were variable in terms of flavonoid and phenolic acid content. Extracts obtained from propolis from southern Poland (EEP1) had the highest phenolic compound content, in particular of flavonoids, and extracts from the remaining propolis (EEP2–EEP5) contained lower amounts of phytochemicals. The quantitative content of propolis is heterogeneous, and the observed differences stem from the variable availability of plants in the field of resin collection by bees [38,39]. Therefore, the majority of propolis are of mixed origin and they often contain components from several plant species [33]. Our study identified for the first time: catechin, apiin, and oroxylin A and cichoric acid, vanillic acid, and syringic acid in samples of Polish propolis. 

The obtained values of DPPH and ABTS scavenging capabilities (IC_50_) by propolis extracts show that they had strong antioxidative activity and high content of total polyphenols. Our results are in line with previous studies, which demonstrated strong correlations between polyphenols content in propolis extracts and their antioxidative activity [40,41,42]. 

Extracts of Polish propolis demonstrated antibacterial activity on test foodborne pathogens. AL-Ani et al. [43] demonstrated similar bacteriostatic activity of propolis from Ireland, Germany, and Czech Republic toward Gram-positive bacteria ranging from 0.08 to 5 mg/mL. Furthermore, our study complies with other observations on the high sensitivity of *S. aureus*, including methicillin-resistant *S. aureus*, to propolis extracts from various parts of the world [44,45,46]. We studied bactericidal EEPs activity toward Gram-negative bacteria, and the MIC remained in the range of 2–16 mg/mL. This observation complies with several other studies in which Gram-negative bacteria have been more resistant than Gram-positive bacteria to the effect of EEP [43,47]. 

The results indicate that EEP1 has higher antibacterial activity, whereas EEP1 and EEP2 have higher antifungal activity than the remaining propolis extracts. This trait may stem from the higher content of phenolic acids, including caffeic acid and flavonoids, in particular pinostrobin, pinobanksin, pinocembrin, galangin, kaempferol, and chrysin, i.e., propolis phytochemicals with antimicrobial activity [38,48]. The antimicrobial properties of flavonoids stem from their capacity to inhibit growth of microorganisms through activation of adhesins, transport proteins of cell membranes. Furthermore, lipophilic flavonoids are capable of rupturing mitochondrial membranes [49]. Despite these observations, we agree with the opinion of other researchers who believe that regardless of the vast chemical diversity of propolis compositions, there is no one single chemical compound or a specific group of compounds that can be associated with antimicrobial activities of propolis extracts, and various flavonoid combinations are necessary for those effects to occur [48,50].

## 4. Materials and Methods 

### 4.1. Propolis Samples

Propolis samples were obtained in the hive from five ecologically clean regions of Poland as indicated in Figure 4. They were characterized by brown color with different shades and color inclusions, typically yellow. These samples originated from Greater Poland Voivodeship and Lesser Poland Voivodeship in southern Poland (Bochnia County; 49°58.143′N; 20°25.8168′E), Masovian Voivodeship in east-central Poland (Siedlce County; 52°10.0632′N; 22°17.4036′E), Łódź Voivodeship in south-east Poland (Opoczno County; 51°22.5414′N; 20°16.6962′E), Podlaskie Voivodeship in north-eastern Poland (Białystok County; 53°7.9998′N; 23°9.8598′E), and Warmian-Masurian Voivodeship in northern Poland (Ełk County; 53°49.6944′N; 22°21.8814′E). Samples were collected during spring–summer of 2017 and were deposited at the Department of Biotechnology, Microbiology and Food Evaluation at the Faculty of Food Sciences of the Warsaw University of Life Sciences–SGGW in Poland. Raw samples of propolis were frozen (−20 °C) and mechanically ground. 

### 4.2. Propolis Extraction Method

Samples (100 g) of pulverized crude propolis were extracted with a 10-fold volume of 70% ethanol solution. Samples were shaken (200 rpm) at 28 °C for 1 day (SM-30 Control, Edmund Bühler, Germany). Subsequently, the samples were subjected to ultrasound and were treated with an Omni Ruptor 4000 sonicator provided by a titanium microtip of diameter 3.8 mm (OMNI International, The Homogenizer Company, Kennesaw, GA, USA). The sonication process was performed for 20 min at a power of 210 W and a frequency of 20 kHz. To prevent excessive heating the samples were immediately placed in ice and water baths. The obtained dry extracts were filtered using gravity filtration on a Whatman No. 4 filter and then condensed under reduced pressure at 40 °C (Rotavapor R-215, Büchi, Switzerland). The EEP were concentrated to dryness by evaporation of the solvent, and then the working solutions were prepared in 70% ethanol. Samples were stored at 4 °C [43,51,52,53].

### 4.3. Analysis of Ethanol Extracts of Propolis (EEP)

#### 4.3.1. Determination of Total Phenolic Content

The total phenolic content was determined by the modified Folin–Ciocalteau method [54,55]: 3 mL EEP was boiled for 3 min and cooled to room temperature. EEP (0.15 mL) was transferred to test tubes and 4.1 mL distilled water and 0.25 mL Folin–Ciocalteau reagent were added. The samples were thoroughly stirred, and after 3 min 1 mL sodium carbonate at 17.7% concentration was added and it was placed in a dark site for 1 h. Subsequently, the solution absorbance was measured against a blank sample (without EEP) at λ 750 nm in a spectrophotometer (Helios Thermo Electron v. 7.03, Thermo Fisher Scientific, Waltham, MA, USA). The polyphenol content was read using a standard curve (y = 55.80x + 0.0155; R^2^ = 0.9968, concentration of gallic acid ranged from 0.0010–0.02 mg/mL) of the dependency of absorbance on polyphenols content, converted into gallic acid. The data were expressed as gallic acid equivalent (GAE) per g of propolis extract.

#### 4.3.2. Determination of Total Flavonoid Content (TFC)

TFC was measured by the aluminum ion chromogenic method with modifications [43,56]: 150 µL of EEP (0.4 mg/mL) was mixed with 2% (*w*/*w*) AlCl_3_ (100 µL) in a 96-well microplate, and then incubated at 37 °C for 30 min; the absorbance at 415 nm was recorded with a Multiskan Sky Microplate Spectrophotometer (Thermo Fisher Scientific, Waltham, MA, USA) microplate reader against a blank (a sample without aluminum chloride). Quercetin standard solutions (0–10 mg/mL) were used for constructing the calibration curve (y = 0.3504x + 0.1514; R^2^ = 0.9936). The data were expressed as quercetin equivalent (QE) per g of propolis extract.

#### 4.3.3. Determination of Antioxidative Capacity Using Synthetic DDPH Radical 

EEP was added to test tubes (20, 40, 80, 125, 150 µL) and supplemented to 2 mL with 80% ethanol, followed by the addition of 2 mL DPPH solution. After mixing, the samples were stored in the dark for 30 min. The absorbance was measured using a spectrophotometer (Helios γ Thermo Spectronic v.7.03) at λ 515 nm, against a blank sample (80% ethanol). The results were expressed as IC_50_ (µg/mL, the concentration of scavenging 50% DPPH radical) [55].

#### 4.3.4. Determination of Antioxidative Capacity in the Reaction of ABTS Radical Scavenging

EEP was added to test tubes (20, 40, 80 µL) along with 3 mL of ABTS radical solution. After mixing, the samples were stored in the dark for 6 min. The absorbance was measured using a spectrophotometer (Helios γ Thermo Spectronic v.7.03) at λ 750 nm, against a blank sample (80% ethanol). The results were expressed as IC_50_ (µg/mL, the concentration of scavenging 50% ABTS radical) [57].

### 4.4. High Performance Liquid Chromatography–Diode Array Detector (HPLC-DAD) Analysis Parameters

#### 4.4.1. Validation

The standards were purchased from Sigma Life Science (Merck, Darmstadt, Germany) and ChromaDex^®^ (Irvine, CA, USA) and separately dissolved with MeOH in a 25-mL volumetric flask according to the ChromaDex’s Tech Tip 0003, Reference Standard Recovery and Dilution, and used as standard stock solutions [58]. Working solutions were prepared by diluting 10 and 100 µL of standard stock solutions with methanol in 10-mL volumetric flasks, 500 and 1000 µL in 5-mL volumetric flasks, as well as 1000 µL in 2-mL volumetric flasks. The working solutions and undiluted stock solutions were injected (1 μL) on a column in six replicates (*n* = 6) using SIL-20AC HT. Six-point calibration curves were plotted according to the external standard method by correlating concentration with peak area. Curve parameters were calculated with Microsoft Excel 14 (Microsoft Corporation, Redmond, WA, USA). The signal-to-noise ratio (S/N) approach was used to determine the limit of detection (S/N of 3:1) and limit of quantification (S/N of 10:1). The peak table and UV spectra library (190–450 nm) of individual compounds were also created.

#### 4.4.2. Parameters of Separations

The obtained propolis extracts were filtered with Iso-Disc™ Filters PTFE-25-2, diameter 25 mm, pore size 0.20 μm (Supelco Analytical™, Bellefonte, PA, USA) and subjected to HPLC. A Shimadzu Prominence chromatograph equipped with an auto sampler SIL-20AC HT, photodiode array detector SPD-M20A, and LC solution 1.21 SP1 chromatography software (Shimadzu, Kyoto, Japan) were used. Separations were achieved using a 100 × 4.60 mm, C18 reversed-phase column, 2.6-μm particles with solid core and porous outer layer (Kinetex™, Phenomenex^®^, Torrance, CA, USA). Binary gradient of deionized water (WCA R03 DP ECO, COBRABiD Aqua, Warsaw, Poland) adjusted to pH 2 with phosphoric acid (Merck) and filtered with 47-mm nylon membrane filter 0.20 μm (Phenex™, Phenomenex^®^, Torrance, CA, USA) as mobile phase A and MeCN (acetonitrile for HPLC ≥ 99.9%, Merck) as phase B was used as follows: 0 min–12.5% B; 25.0 min–40% B; 34.0 min–60% B; 37.0 min–95% B; 37.1 min–12.5% B; 40 min–stop. The flow rate was set to 2.0 mL/min, oven temperature 45 °C, injection volume 1 μL. Peak identification was carried out by comparison of retention time as well UV spectra with standards. The content of the determined compounds was calculated in mg per 100 mL of propolis extract. 

### 4.5. Test Microorganisms and Preparation of Inoculum

Reference strains were obtained from the American Type Culture Collection (ATCC, Manassas, VA, USA), Institute of Plant Protection–National Research Institute (IOR, Poznań, Poland), Collection of Industrial Microorganisms (KKP, Warsaw, Poland), and Leibniz Institute DSMZ–German Collection of Microorganisms and Cell Cultures (DSMZ, Braunschweig, Germany) and the clinical strain was provided by the National Institute of Public Health–National Institute of Hygiene (NIPH-NIH, Warsaw, Poland). The study used four strains of Gram-positive bacteria (*Staphylococcus aureus* ATCC 25923, *Bacillus cereus* ATCC 11778, *Listeria monocytogenes* ATCC 7644, *Enterococcus faecalis* ATCC 29212), seven strains of Gram-negative bacteria (*Salmonella* Enteritidis ATCC 13076, *Shigella sonnei* NIPH-NIH “s”, *Klebsiella pneumoniae* ATCC 13883, *Escherichia coli* O157 ATCC 700728, *Proteus mirabilis* ATCC 35659, *Enterobacter aerogenes* ATCC 13048, *Pseudomonas aeruginosa* ATCC 27853), four strains of yeast *(Rhodotorula mucilaginosa* ATCC 66034, *Candida albicans* ATCC 10231, *Candida krusei* ATCC 14243, *Saccharomyces cerevisiae* ATCC 9763), and 11 strains of molds (*Colletotrichum gloeosporioides* DSMZ 62146, *Alternaria solani* ATCC 16022, *Fusarium solani* ATCC 36031, *Rhizopus stolonifer* ATCC 14037, *Botrytis cinerea* IOR 2110, *Cladosporium cladosporioides* ATCC 16022, *Aspergillus niger* ATCC 9142, *Aspergillus ochraceus* KKP 124, *Mucor mucedo* ATCC 38694, *Penicillium expansum* KKP 774, *Penicillium chrysogenum* ATCC 10136). 

The bacterial strains were cultured on Müller–Hinton Agar (MHA; Merck) and incubated at 37 °C ± 1 °C for 24 h. Bacterial inocula were prepared in sterile 0.85% NaCl (*w*/*v*) solution to reach a population of approximately 1 × 10^8^ CFU/mL. The yeast strains were grown on Sabourand Agar (SA; Merck) at 28 °C for 24 h. The mold spores were obtained from mycelium grown on SA after incubation at 22 °C for 14 days. Fungal suspensions were prepared in sterile 0.85% NaCl to achieve 10^6^ spores/mL. The number of yeast cells and spores was calculated using a hemocytometer. 

### 4.6. Determination of Minimum Inhibitory Concentration (MIC) and Minimum Bactericidal and Fungicidal Concentration (MBC, MFC)

The MIC and MBC/MFC of EEP were determined using the serial microdilution method (CLSI, 2009). 

Dilutions of EEPs were prepared in Müller–Hinton Broth (MHB; Merck) and RPMI 1640 medium (Merck) in the concentration range of 320–5 mg/mL. Then, 20 μL of EEP from each dilution and 10 μL of bacterial/fungal suspensions were separately transferred to 96-well plates. Each well contained 170 μL of MHB or RPMI 1640. The final volume of each well was 200 μL and the final EEP concentration was in the range of 32–0.5 mg/mL. The final concentration of the bacterial inoculum was 5 × 10^5^ CFU/mL, and that of the yeast and mold was 5 × 10^4^ CFU/mL. The wells containing inoculated and non-inoculated broth were prepared as growth and purity controls simultaneously. The plates with bacteria were incubated at 37 °C for 24 h. Plates with yeasts were incubated at 28 °C for 48 h and with molds at 22 °C for 72 h. The MIC value was defined as the lowest concentration of EEP in which no visual growth of bacteria or fungi compared with the EEP sample-free control was found, and was expressed in mg/mL. The MIC examination of EEP was repeated three times. 

In order to determine the MBC and MFC concentrations of propolis extract, 100 μL of bacterial/fungal culture from each well, where no growth was observed, was reinoculated onto MHA (for bacteria) or SA (for fungi) plates, which were incubated at 37 °C for 24 h (for bacteria) or at 28 °C for 72 h (for fungi). The plates were checked for growth of colonies. The MBC/MFC was defined as the lowest concentration of propolis extract which resulted in complete inhibition of bacterial/fungal growth, and was expressed in mg/mL.

### 4.7. Percentage Value of Antimicrobial Activity

The percentage value of antimicrobial activity of EEP was determined based on MIC values (A) [59]:

A (%) = (100 × number of strains inhibited by the examined preparation)/(total number of tested strains)(1)

The percentage of activity denotes the complete antimicrobial potency of an EEP, i.e., it determines the number of bacterial strains susceptible to one specific EEP.

### 4.8. Time-Kill Assay

A time-kill kinetics assay was performed according to AL-Ani et al. [43]. Briefly, tubes containing MHB or RPMI-1640 broth with 1 × MIC concentration of EEPs were incubated at 37 °C with 5 × 10^7^ CFU/mL of bacterial suspension or at 28 °C with 5 × 10^7^ CFU/mL of fungal suspensions. Then, 100-µL aliquots were removed from all tubes after the incubation period at 0, 2, 4, 6, and 24 h (and 48 h for yeast) and 10-fold serial dilution was prepared with normal saline and 20-µL aliquots from dilutions were plated by MHA or SA and incubated at the optimal temperature for 18–24 h. The results were expressed in CFU/mL.

### 4.9. Statistical Analysis

Data were presented as mean ± SD. Three replications were carried out for each experiment. Statistical tests were performed using the Statistica version 13 PL computer program (TIBCO, Palo Alto, CA, USA). Multivariate correlation analysis was used for the evaluation of the spectrum–effect relationships. One-way analysis of variance was carried out. The significance of differences between mean values was assessed using Tukey test at a significance level of *p* < 0.05.

## 5. Conclusions

The present study examined the chemical composition, and antioxidative and antimicrobial properties of Polish propolis originating from five typical agricultural regions of Poland. All EEPs contained flavonoids and phenolic acids; yet their amounts were variable. All propolis extracts inhibited growth of foodborne bacteria and fungi contaminating food. The EEP originating from south Poland was distinguished by higher antimicrobial activity and at the same time higher polyphenols content relative to the remaining propolis extracts. Extracts from Polish propolis can be taken into consideration for the biocontrol of foodborne pathogens.

## Figures and Tables

**Table 1:** HPLC-DAD (High Performance Liquid Chromatography–Diode Array Detector) validation parameters (*n* = 6).

No.	Compound	Precision Intra-Day (CV %)	Precision Inter-Day (CV %)	Calibration Equation	R^2^ (*n* = 6)	Linear Range (mg/mL)	LOD (µg/L)	LOQ (µg/L)
1	Protocatechuic acid	0.30	0.80	y = 7102.90 x + 43850.00	0.9998	0.38–380.00	6.69	22.28
2	(+)-Catechin	0.34	1.21	y = 8216.40x − 6069.30	0.9998	0.95–950.00	10.90	36.40
3	4-Hydroxybenzoic acid	0.86	1.25	y = 4563.72 x + 2195.48	0.9999	1.06–1059.30	7.21	24.02
4	Caffeic acid	1.00	1.72	y = 2592.90 x + 379.64	0.9996	0.998–998.40	2.50	8.32
5	Vanillic acid	1.14	1.88	y = 2854.67 x − 1567.35	0.9999	1.14–1144.60	21.04	70.14
6	Syringic acid	0.65	0.98	y = 5762.99 x − 264.13	0.9999	0.39–392.00	6.40	21.32
7	*p*-Coumaric acid	0.28	0.65	y = 6196.40 x − 537.45	0.9999	1.01–504.70	6.87	22.90
8	Ferulic acid	0.58	0.84	y = 2424.60 x − 1856.88	0.9995	0.40–399.68	10.57	35.23
9	Ellagic acid dihydrate	1.05	2.08	y = 8108.60 x + 3974.20	0.9999	1.00–999.88	6.50	20.16
10	Quercetin 3-*O*-rutinoside	0.37	0.86	y = 1434.00 x − 5093.00	0.9999	0.91–90.67	7.46	24.88
11	Cichoric acid	0.18	0.49	y = 3230.70 x + 6882.20	0.9998	0.46–456.96	11.47	38.23
12	Apiin	0.88	1.27	y = 2757.84 x − 1062.55	0.9999	0.69–690.12	12.12	40.41
13	Dimethyl caffeic acid	0.70	0.91	y = 2539.82 x + 7831.69	0.9997	0.84–839.80	12.46	41.53
14	Cinnamyl alcohol	0.29	0.51	y = 6326.13 x − 298.76	0.9996	1.16–116.40	6.612	22.06
15	Cinnamic acid	0.68	1.01	y = 6875.85 x − 9588.37	0.9996	1.15–115.43	6.14	20.48
16	4-Methoxycinnamic acid	0.57	0.84	y = 4155.62 x − 9125.38	0.9998	1.16–577.60	8.77	29.24
17	Quercetin	0.52	0.89	y = 2260.21 x + 744.64	0.9999	0.41–408.34	4.22	14.05
18	Pinobanksin	0.42	0.75	y = 2259.79 x − 898.10	0.9999	0.19–192.85	16.30	54.33
19	Apigenin	0.21	0.57	y = 1994.70 x + 1248.70	0.9999	0.38–377.88	15.58	51.93
20	Kaempferol	0.10	0.45	y = 2064.66 x − 176.08	0.9999	0.47–469.00	19.34	64.45
21	Isorhamnetin	1.28	1.85	y = 4004.00 x − 7104.50	0.9997	0.398–397.99	15.80	52.80
22	Chrysin	0.58	0.89	y = 4160.14 x + 39.94	0.9999	0.35–351.50	8.00	26.65
23	Pinocembrin	0.21	0.32	y = 2632.71 x + 2230.68	0.9999	0.99–988.00	18.30	61.00
24	Acacetin	1.27	1.48	y = 2673.25 x + 4227.20	0.9998	0.38–383.60	10.14	33.81
25	Galangin	0.26	0.45	y = 3097.03 x − 6549.41	0.9999	0.95–951.52	15.73	52.42
26	Oroxylin A	1.16	1.55	y = 929.85 x − 623.61	0.9999	1.34–1335.34	86.60	288.67
27	(+/−)-Pinostrobin	0.28	0.38	y = 2810.87 x + 3430.58	0.9998	0.52–517.77	12.99	43.31

**Table 2:** The chemical composition of ethanol extracts of propolis (EEP) [mg/100 mL].

	CAS	Compound	EEP1	EEP2	EEP3	EEP4	EEP5
1	99-50-3	Protocatechuic acid	14.57 ± 0.02	15.00 ± 0.10	20.09 ± 0.12	15.55 ± 0.10	14.14 ± 0.16
2	154-23-4	(+)-Catechin	29.23 ± 0.70	6.77 ± 0.02	12.32 ± 0.13	4.05 ± 0.15	5.64 ± 0.08
3	99-96-7	4-Hydroxybenzoic acid	48.68 ± 0.33	164.64 ± 3.28	250.14 ± 8.15	176.49 ± 7.73	173.12 ± 6.00
4	331-89-5	Caffeic acid	1452.74 ± 24.37	384.09 ± 4.07	453.30 ± 3.48	286.95 ± 6.42	432.55 ± 2.62
5	121-34-6	Vanillic acid	25.65 ± 0.39	67.45 ± 0.55	99.21 ± 1.07	99.83 ± 0.69	68.64 ± 0.93
6	530-57-4	Syringic acid	3.02 ± 0.03	3.44 ± 0.02	4.95 ± 0.01	3.24 ± 0.03	3.45 ± 0.09
7	501-98-4	*p*-Coumaric acid	1269.99 ± 45.35	1912.61 ± 19.84	2766.97 ± 28.00	2550.03 ± 27.06	1959.47 ± 28.05
8	1135-24-6	Ferulic acid	610.87 ± 5.53	1525.48 ± 26.66	2418.23 ± 31.40	2067.11 ± 51.45	1775.29 ± 21.40
9	476-66-4	Ellagic acid dihydrate	0.00 ± 0.00	0.00 ± 0.00	4.71 ± 0.10	2.03 ± 0.02	1.36 ± 0.01
10	153-18-4	Quercetin 3-*O*-rutinoside	0.00 ± 0.00	0.00 ± 0.00	81.10 ± 1.50	8.38 ± 0.11	10.35 ± 0.57
11	6537-80-0	Cichoric acid	23.14 ± 0.56	110.71 ± 2.17	116.41 ± 1.76	172.51 ± 4.02	108.48 ± 2.16
12	26544-34-3	Apiin	5.02 ± 0.07	0.00 ± 0.00	0.00 ± 0.00	0.00 ± 0.00	0.00 ± 0.00
13	2316-26-9	Dimethyl caffeic acid	973.45 ± 18.33	119.14 ± 2.26	116.99 ± 1.11	78.89 ± 3.48	234.71 ± 5.27
14	104-54-1	Cinnamyl alcohol	44.63 ± 1.05	0.00 ± 0.00	0.00 ± 0.00	5.17 ± 0.13	0.00 ± 0.00
15	140-10-3	Cinnamic acid	93.79 ± 2.05	45.63 ± 1.18	60.23 ± 0.73	48.53 ± 0.73	48.69 ± 1.93
16	943-89-5	4-Methoxycinnamic acid	61.30 ± 1.53	23.33 ± 0.24	15.62 ± 0.51	2.12 ± 0.19	50.13 ± 2.87
17	117-39-5	Quercetin	189.64 ± 3.85	79.06 ± 0.54	39.80 ± 0.68	27.74 ± 0.97	52.70 ± 0.27
18	548-82-3	Pinobanksin	657.94 ± 4.10	285.60 ± 5.09	254.07 ± 2.12	198.86 ± 4.44	283.73 ± 6.52
19	520-36-5	Apigenin	640.41 ± 8.18	282.52 ± 6.91	209.54 ± 7.74	201.01 ± 4.85	238.07 ± 5.49
20	520-18-3	Kaempferol	419.10 ± 4.74	184.30 ± 4.31	111.89 ± 3.32	129.04 ± 4.91	151.98 ± 1.87
21	480-19-3	Isorhamnetin	48.65 ± 0.65	50.82 ± 0.31	10.75 ± 0.15	29.16 ± 0.48	33.61 ± 0.53
22	480-40-0	Chrysin	5766.55 ± 152.14	994.83 ± 7.52	533.32 ± 5.60	343.91 ± 5.69	1046.65 ± 29.28
23	480-39-7	Pinocembrin	1626.32 ± 39.96	1111.27 ± 17.88	407.40 ± 12.02	588.16 ± 11.89	907.82 ± 14.80
24	480-44-4	Acacetin	0.00 ± 0.00	0.00 ± 0.00	0.00 ± 0.00	246.82 ± 6.96	214.15 ± 2.69
25	548-83-4	Galangin	664.26 ± 3.82	353.08 ± 4.70	9.52 ± 0.50	184.24 ± 4.21	405.70 ± 3.94
26	480-11-5	Oroxylin A	74.93 ± 1.11	87.13 ± 1.65	33.18 ± 1.05	79.27 ± 3.08	73.33 ± 0.75
27	480-37-5	(+/−)-Pinostrobin	74.06 ± 0.65	1029.32 ± 9.31	50.21 ± 0.61	164.09 ± 2.28	281.38 ± 3.74
		Total alcohol	44.63	0.00	0.00	5.17	0.00
		Total flavan-3-ols	29.23	6.77	12.32	4.05	5.64
		Total flavanones	74.06	1029.32	50.21	164.09	281.38
		Total flavanonols	2284.26	1396.86	661.46	787.03	1191.55
		Total flavones	6486.90	1364.48	776.03	871.01	1572.20
		Total flavonols	1321.65	667.25	253.07	378.56	654.34
		Total phenolic acids	4577.21	4371.53	6326.85	5503.29	4870.02
		Total flavonoids	10196.09	4464.69	1753.09	2204.74	3705.10
		Total phenolics	14773.30	8836.22	8079.94	7708.03	8575.12

**Table 3:** The results of total flavonoid content (TFC), total polyphenols content (TPC) and antioxidant activity analyses in vitro (DPPH, ABTS).

	TFC (mg QE/g)	TPC (mg GAE/g)	DPPH Scavenging Activity (IC_50_, µg/mL)	ABTS Scavenging Activity (IC_50_, µg/mL)
EEP1	15.55 ± 0.35 ^e^	100.29 ± 1.03 ^c^	0.93 ± 0.07 ^a^	0.36 ± 0.05 ^a^
EEP2	14.58 ± 0.23 ^d^	52.93 ± 2.08 ^a^	2.07 ± 0.11 ^c^	0.60 ± 0.17 ^b^
EEP3	8.23 ± 0.15 ^a^	68.46 ± 1.72 ^b^	1.67 ± 0.05 ^b^	0.31 ± 0.12 ^a^
EEP4	9.98 ± 0.15 ^b^	54.98 ± 1.15 ^a^	2.08 ± 0.09 ^c^	0.51 ± 0.13 ^a,b^
EEP5	13.30 ± 0.13 ^c^	52.65 ± 3.01 ^a^	1.87 ± 0.03 ^b^	0.33 ± 0.07 ^a^

TFC‒Total Flavonoid Content, TPC‒Total Polyphenols Content, Means marked in column with different letters (a–e) differ at *p* < 0.05.

**Table 4:** Antibacterial activity of ethanol extracts of propolis.

Strain	EEP1	EEP2	EEP3	EEP4	EEP5
	MIC (MBC) (mg/mL)				
**Gram (+) Bacteria**					
*Staphylococcus aureus*	1 (4)	8 (16)	2 (8)	2 (8)	2 (8)
*Bacillus cereus*	2 (32)	4 (32)	2 (32)	4 (32)	4 (32)
*Listeria monocytogenes*	4 (8)	4 (8)	8 (16)	8 (16)	8 (16)
*Enterococcus faecalis*	4 (8)	8 (16)	8 (16)	8 (16)	8 (16)
**Gram (−) Bacteria**					
*Salmonella* Enteritidis	16 (16)	8 (16)	8 (16)	16 (32)	16 (32)
*Shigella sonnei*	4 (8)	8 (16)	8 (16)	16 (32)	8 (16)
*Klebsiella pneumoniae*	2 (8)	8 (16)	16 (32)	16 (32)	16 (32)
*Escherichia coli* O157	16 (16)	16 (32)	16 (32)	16 (32)	16 (32)
*Proteus mirabilis*	8 (16)	8 (16)	8 (16)	8 (16)	8 (16)
*Enterobacter aerogenes*	8 (16)	16 (32)	8 (16)	8 (16)	16 (32)
*Pseudomonas aeruginosa*	8 (16)	8 (16)	16 (16)	8 (16)	8 (16)

MIC–Minimum Inhibitory Concentration; MBC–Minimum Bactericidal Concentration.

**Table 5:** Percentage of antibacterial activity (A %).

MIC (mg/mL)	EEP1	EEP2	EEP3	EEP4	EEP5
0.5	0	0	0	0	0
1	9	0	0	0	0
2	27	0	18	9	9
4	55	18	18	18	18
8	82	82	73	64	64
16	100	100	100	100	100
32	100	100	100	100	100

MIC–Minimum Inhibitory Concentration.

**Table 6:** Antifungal activity of ethanol extracts of propolis.

	EEP1	EEP2	EEP3	EEP4	EEP5
	MIC (MFC) (mg/mL)				
**Yeast**					
*Rhodotorula mucilaginosa*	4 (8)	4 (8)	8 (16)	8 (16)	4 (16)
*Candida albicans*	2 (4)	4 (8)	32 (32)	16 (32)	16 (16)
*Candida krusei*	4 (8)	4 (16)	16 (32)	16 (32)	8 (16)
*Saccharomyces cerevisiae*	4 (8)	4 (8)	16 (32)	8 (16)	4 (16)
**Mold**					
*Colletotrichum gloeosporoides*	4 (4)	2 (2)	4 (4)	4 (8)	2 (2)
*Alternaria solani*	4 (4)	2 (4)	8 (8)	8 (8)	4 (4)
*Fusarium solani*	2 (4)	4 (8)	4 (16)	4 (8)	4 (4)
*Rhizopus stolonifer*	4 (32)	4 (32)	8 (32)	4 (32)	4 (32)
*Botrytis cinerea*	4 (4)	4 (4)	8 (8)	4 (4)	4 (4)
*Cladosporium cladosporoides*	8 (8)	4 (8)	8 (16)	8 (16)	4 (8)
*Aspergillus niger*	8 (16)	4 (8)	32 (32)	32 (32)	32 (32)
*Aspergillus ochraceus*	8 (8)	8 (8)	16 (16)	16 (16)	8 (8)
*Mucor mucedo*	8 (8)	4 (4)	8 (8)	8 (8)	4 (8)
*Penicillium expansum*	8 (16)	8 (16)	8 (16)	8 (16)	8 (16)
*Penicillium chrysogenum*	16 (32)	8 (16)	16 (32)	16 (32)	16 (32)

MIC–Minimum Inhibitory Concentration; MFC–Minimum Fungicidal Concentration.

**Table 7:** Percentage of antifungal activity (A %).

MIC (mg/mL)	EEP 1	EEP 2	EEP 3	EEP 4	EEP 5
0.5	0	0	0	0	0
1	0	0	0	0	0
2	13	13	0	0	7
4	60	80	13	27	60
8	93	100	60	67	80
16	100	100	87	93	93
32	100	100	100	100	100

MIC–Minimum Inhibitory Concentration.
